# Prevention and management of hypertensive crises in children with pheochromocytoma and paraganglioma

**DOI:** 10.3389/fendo.2024.1460320

**Published:** 2024-08-20

**Authors:** Chiara Bima, Chiara Lopez, Gerdi Tuli, Jessica Munarin, Stefano Arata, Matteo Procopio, Martina Bollati, Mauro Maccario, Luisa De Sanctis, Mirko Parasiliti-Caprino

**Affiliations:** ^1^ Endocrinology, Diabetes and Metabolism; Department of Medical Sciences; University of Turin, Turin, Italy; ^2^ Department of Sciences of Public Health and Pediatrics, University of Turin, Turin, Italy

**Keywords:** juvenile hypertension, catecholamines, pheochromocytoma, paraganglioma, secondary hypertension, hypertensive crisis, children

## Abstract

Hypertensive crises in pediatric patients are rare conditions. However, determining their precise prevalence is more challenging than in adults due to the heterogeneity in the definition itself. These crises frequently occur without a prior diagnosis of hypertension and may indicate an underlying cause of secondary hypertension, including pheochromocytoma/paraganglioma (PPGL). The mechanisms of hypertensive crises in the pediatric population with PPGL are directly related to different types of catecholamine excess. Noradrenergic tumors typically present with sustained hypertension due to their predominant action on α1-adrenoceptors in the vasculature. Conversely, adrenergic tumors, through epinephrine binding to β2-adrenoceptors in addition to stimulation of α1- and α2-adrenoceptors, more frequently cause paroxysmal hypertension. Furthermore, the biochemical phenotype also reflects the tumor localization and the presence of a genetic mutation. Recent evidence suggests that more than 80% of PPGL in pediatric cases have a hereditary background. PPGL susceptibility mutations are categorized into three clusters; mutations in cluster 1 are more frequently associated with a noradrenergic phenotype, whereas those in cluster 2 are associated with an adrenergic phenotype. Consequently, the treatment of hypertensive crises in pediatric patients with PPGL, reflecting the underlying pathophysiology, requires first-line therapy with alpha-blockers, potentially in combination with beta-blockers only in the case of tachyarrhythmia after adequate alpha-blockade. The route of administration for treatment depends on the context, such as intraoperative or pre-surgical settings, and whether it presents as a hypertensive emergency (elevated blood pressure with acute target organ damage), where intravenous administration of antihypertensive drugs is mandatory. Conversely, in cases of hypertensive urgency, if children can tolerate oral therapy, intravenous administration may initially be avoided. However, managing these cases is complex and requires careful consideration of the selection and timing of therapy administration, particularly in pediatric patients. Therefore, facing these conditions in tertiary care centers through interdisciplinary collaboration is advisable to optimize therapeutic outcomes.

## Introduction and epidemiology

The prevalence of arterial hypertension in pediatric patients varies from 0.5-1% to 5% depending on the studies considered (1, 2). However, determining the prevalence of hypertensive crises in children is more challenging due to the heterogeneity in its definition. Generally, a hypertensive crisis in pediatric patients is suspected when blood pressure exceeds the limits of stage II hypertension (3). Specifically, the European Society of Hypertension (ESH) guidelines define severe hypertensive crisis in children as blood pressure values 20% above the stage II hypertension limit (4). Hypertensive crises can be categorized as hypertensive urgency and emergency, with the latter defined by elevated blood pressure values associated with acute target organ damage (2).

In adults, hypertensive crises are often associated with poor compliance with antihypertensive treatment. While in children, they frequently occur without a known previous diagnosis of hypertension and may reflect an underlying cause of secondary hypertension, predominantly of renal origin (70-80% of cases), but also of an endocrine one, including the diagnosis of pheochromocytoma/paraganglioma (PPGL) (3, 5). PPGLs are rare neuroendocrine tumors that can secrete catecholamines (in about the 80% of cases), arising from chromaffin cells in the adrenal medulla or sympathetic paraganglia. The incidence of PPGL is approximately 1 in 300,000 per year, with only 10-20% occurring in pediatric age, with an estimated incidence of 0.5-2 per million children, with pheochromocytomas (PCC) representing 80-85% of cases compared to paragangliomas (PGL) (6–8). A retrospective study by Pamporaki et al. (9) showed a high percentage of hereditary (up to 70-80%), extra-adrenal, metastatic, multifocal, and recurrent disease in pediatric-onset tumors (10).

Among children with arterial hypertension, the incidence of PPGL is high, around 1.7%, compared to 0.2-0.6% in hypertensive adults (8, 10). Clinical presentation varies, with signs and symptoms of catecholamine hypersecretion often overlooked in children due to their high level of physical activity compared to adults. Children with PPGL are more likely to have sustained arterial hypertension, up to 60-90% of them, while adults often exhibit paroxysmal hypertension in about 50% of cases (5, 8, 10). The clinical presentation of PPGL reflects the underlying catecholamine secretory phenotype and genotype, with epinephrine-secreting tumors more associated with an acute and explosive presentation than norepinephrine and dopamine-secreting lesions (10).

Treating arterial hypertension, particularly hypertensive crises, in children presents significant challenges. Given the rarity of PPGL in pediatric patients and the limited data available in the literature, the objective of this review is to provide guidance for the clinical management of PPGL-induced hypertensive crises in children. This includes advocating for interdisciplinary collaboration among endocrinologists, pediatric intensivists, anesthesiologists, nephrologists, emergency physicians and surgeons to ensure comprehensive care.

## Pathophysiology

The mechanisms of hypertensive crisis in pediatric population with PPGL is directly related to the different types of catecholamine excess (10, 11). From a physiological point of view, norepinephrine and epinephrine are released in different pattern from chromaffin cells tumors and show variable binding affinities for adrenoreceptors, leading to different clinical manifestations as the results of their effect on hemodynamics and metabolism (11). Approximately 50% of pheochromocytomas produce norepinephrine almost exclusively, whereas the other half secretes a combination of norepinephrine and epinephrine.

Adrenoreceptors are G-protein coupled receptor mediating the actions of epinephrine and norepinephrine. The major types of human adrenoreceptors are: α1, α2 and β, each having more subtypes. α1-adrenoceptors are mainly located in the vasculature and their stimulation induces vasoconstriction and increased peripheral vascular resistances, resulting in sustained hypertension. Instead, stimulation of presynaptic α2-adrenoceptors can reduce neuronal norepinephrine release, decreasing blood pressure, through a negative feedback mechanism. β1-adrenoceptors, localized on cardiomyocytes and the cardiac conduction system, mediate increase in blood pressure, through their action on heart rate and cardiac output. In contrast, stimulation of β2-adrenoceptors, present both on blood vessels and cardiomyocytes, could induce vasodilatation and reduced cardiac inotropy, and consequently hypotension (12). Norepinephrine exerts its cardiovascular effects by working mainly on α1-adrenoceptors in the vasculature, with low activity on cardiac β1-adrenoceptors. Instead, epinephrine is responsible for its hemodynamic and metabolic actions through its binding to β2-adrenoceptors, in addiction to stimulation of both α1- and α2-adrenoceptors (12).

Form the clinical point of view, the evaluation of catecholamine O-methylated metabolites is of primary importance because their production and release are continuous and independent from catecholamine secretion, making them reliable disease markers. The main metabolites are normetanephrine, metanephrine and 3-methoxytyramine, deriving from norepinephrine, epinephrine and dopamine, respectively (11, 13).

However, regardless of underlying etiology, hypertensive crisis is characterized by some common pathways that could contribute to the severity of hypertension itself and to end-stage organ damage (14). As shown by Harrison et al. (15), the initial stimulus to hypertension could involve several systems, determining the activation of renin-angiotensin-aldosterone system, oxidative stress and endothelial dysfunction. The consequent protein fragmentation and formation of neoantigens result in activation of T-cells and release of cytokines able to increase vasoconstriction and sodium and water retention. This theory thus recognizes a crucial role of inflammation in the pathogenesis of hypertensive crisis and possible organ damage (16).

**Figure 1:**
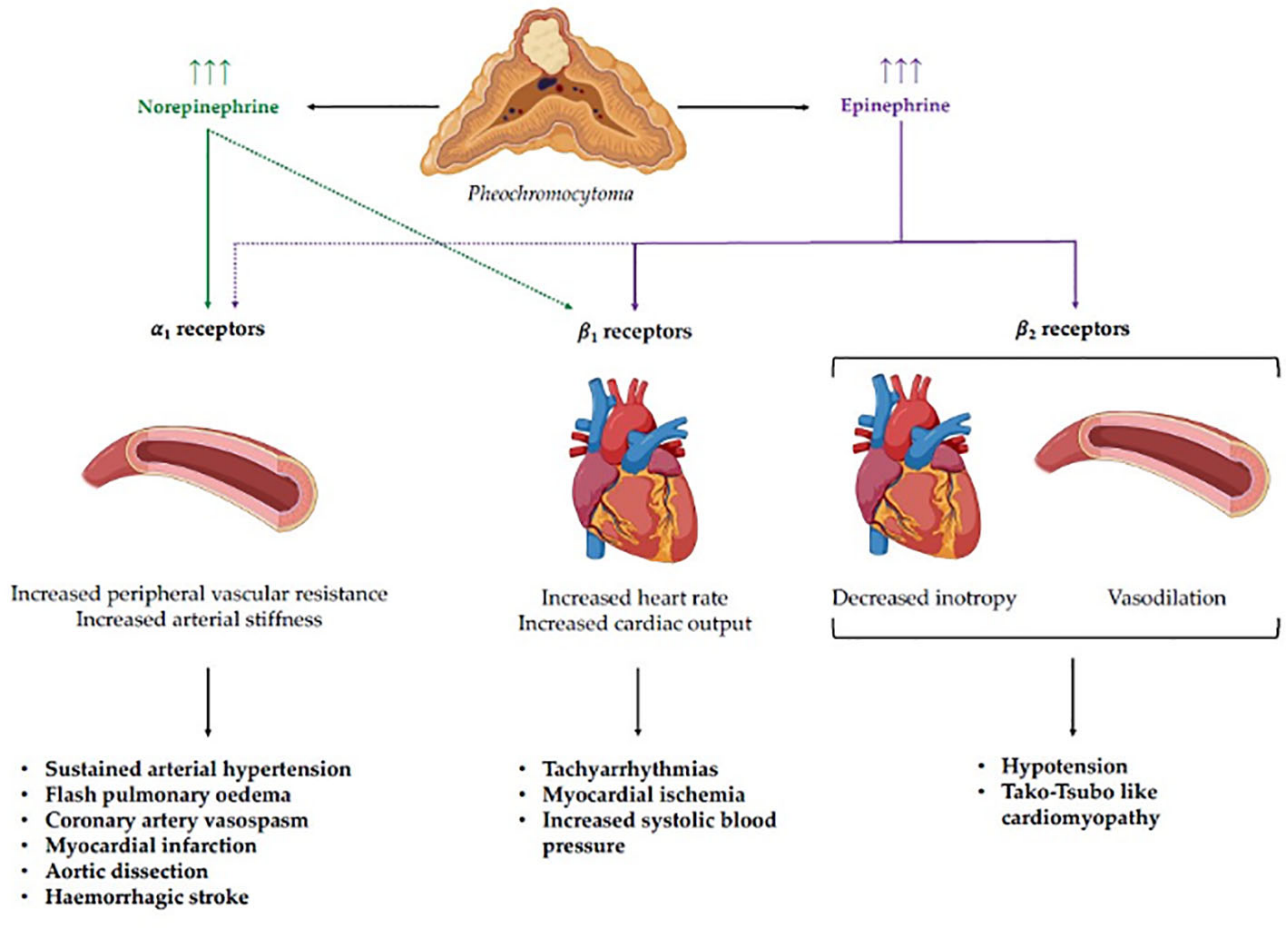
Pathophysiology of catecholamine-induced hypertensive crises and related cardiovascular complications. Norepinephrine and epinephrine display different binding affinities for adreno-receptors, leading to distinct clinical phenotypes according to the specific secreting pattern of the pheochromocytoma. Stimulation of *α*1 receptors, which show a higher affinity for norepinephrine, mediates an increase in peripheral vascular resistance, leading to sustained arterial hypertension and complications such as flash pulmonary edema, coronary artery vasospasm, myocardial infarction, aortic dissection, and hemorrhagic stroke. On the other hand, stimulation of cardiac *β*1 receptors, which have a greater affinity for epinephrine, leads to a positive chronotropic and inotropic effect, with the potential development of tachyarrhythmias, myocardial ischemia, and systolic hypertension. Finally, the stimulation of cardiac and vascular *β*2 receptors by epinephrine can lead to negative inotropism and vasodilation with subsequent hypotension and Tako-Tsubo like cardiomyopathy.

The abovementioned pathophysiology of cathecolamine-induced hypertensive crises and related cardiovascular complications is depicted in **Figure 1**.

## Clinical manifestations and biochemical phenotypes

In childhood functional PPGLs frequently manifest with sustained, rather than paroxysmal, hypertension present in approximately 60-90% of patients (5, 8). Furthermore, the classic “triad” including diaphoresis, headaches, and palpitations has been reported in up to 54% of children with PPGLs (8). In PPGL the cardiovascular complication of hypertensive crisis could derive, apart from marked and abrupt elevation in blood pressure values, also from catecholamine direct damage of the heart and vessels (17). However, the most common manifestations of hypertensive crisis in children are neurologic (14). Clinical signs and symptoms typically depend on type of hormonal secretion (11, 12). The specific secretion profile results in three different biochemical phenotypes: noradrenergic, adrenergic and dopaminergic, according to the predominant increase in normetanephrine, metanephrine and 3-methoxytyramine, respectively (12, 13). Moreover, there is a rare PPGL subtype that does not produce and release catecholamines, named biochemically silent (12). Noradrenergic tumors typically occur with sustained hypertension and possible consequences of excessive α1-adrenoceptors stimulation on cardiovascular system, such as flash pulmonary edema, coronary artery vasospasm and myocardial infarction, or aortic dissection. Instead, adrenergic tumors, in addition to the sequalae of α1-adrenoceptors stimulation, could also manifest more sequelae of excessive β-adrenoceptor stimulation, resulting in tachyarrhythmia, myocarditis, and demand myocardial ischemia or infarction (17, 18). Moreover, because of excessive β2-adrenoceptor binding, adrenergic tumors may also manifest with hypoglycemia, suppression of myocardial contractility (as in Takotsubo-like cardiomyopathy), orthostatic hypotension, and even hypotensive shock (8). Conversely, patients with dopamine-secreting tumors, could be diagnosed by mass effect symptoms, being longer normotensive and asymptomatic (5).

The biochemical phenotype also influences tumor localization. In fact, PPGL with noradrenergic phenotype is typically extra-adrenal instead adrenergic PPGLs are characterized by location within the adrenal glands (12).

## Genetic background

Overall, PPGL are characterized by the highest rate of hereditary background compared to other human neoplasms. In fact, approximately 40–50% of cases are related to germline mutations in one of the known susceptibility genes and in 40-60% of sporadic cases a somatic mutation is found (19–21). Moreover, the hereditary rate rises to 70-80% in the pediatric setting (7, 9, 22). Baush et al. (7) reported an even higher 80% frequency of germline mutations among pediatric cases. Early onset of disease, bilateral multifocal, extra-adrenal, and malignant tumors are the clinical hallmarks of hereditary disease. Moreover, children usually presented symptomatic and potentially life-threatening disease (6).

Specific germline mutations have been attributed to at least 25 tumor-susceptibility genes (19, 20) that could be divided into three cluster groups based on transcriptomic profiles revealed by gene expression microarray analyses (23–26). This proportion of implicated genes will progressively increase as mutations impacting new genes are found (21).

Cluster 1 tumors comprehend those related to the following mutations: von Hippel-Lindau (*VHL*) suppressor, the four subunits of the succinate dehydrogenase complex (*SDHA, SDHB, SDHC*, and *SDHD*), and less commonly, the enzyme responsible for flavination of the SDHA subunit (*SDHAF2*), fumarate hydratase (*FH*), malate dehydrogenase 2 (*MDH2*), prolyl hydroxylase (*PHD*), and somatic gain-of-function pathogenic variants in the hypoxia-inducible factor 2 alpha gene (*HIF2A* or *EPAS1*) and some newly discovered genes that will be detailed in **Table 1**. Cluster 1 mutations result in stabilization of hypoxia-inducible factors and activation of the hypoxia signaling pathways (25, 26).

**Table 1:** Characteristics of the main PPGL-susceptibility genes (5, 8, 12, 19–21, 27).

Gene	Gene type	Most common PPGL location	Biochemical phenotype	Related syndromes and other manifestations	Malignancy risk
Cluster 1 (pseudohypoxic signaling)					
*SDHA*	Germline	sPGL, HNPGL, PCC (very low penetrance)	NE; NE+DA; NS	PGL6 RCC; GIST; pituitary adenoma	0-14%
*SDHB*	Germline	sPGL, HNPGL (intermediate penetrance) PCC (low penetrance)	NE; NE+DA; NS	PGL4 RCC; GIST; pituitary adenoma; pulmonary condroma	34-70%
*SDHC*	Germline	HNPGL, sPGL (intermediate penetrance) PCC (low penetrance)	NS; NE +DA	PGL3 RCC; GIST; pituitary adenoma	0-28%
*SDHD*	Germline	Multifocal HNPGL (high penetrance) sPGL (low penetrance) PCC (low penetrance	NE; NE +DA; NS	PGL1 RCC; GIST; pituitary adenoma; pulmonary condroma	<5%
*SDHAF2*	Germline	Multifocal HNPGL (high penetrance)	NS	PGL2	/
*FH*	Germline	PCC + sPGL	NE	Hereditary Leiomyomatosis and Renal Cell Cancer (HLRCC)	/
*MDH2*	Germline	sPGL	NE	Early-Onset Severe Encephalopathy (homozygous germline mutations)	/
*VHL*	Germline/somatic	PCC (high penetrance) usually bilateral (50%) sPGL (low penetrance) also multifocal HNPGL (very low penetrance)	NE	VHL 2A: retinal and CNS hemangioblastomas, endolymphatic sac tumors, epididymal cystadenomas 2B: renal and pancreatic cell cysts and carcinomas, retinal and CNS hemangioblastomas, endolymphatic sac tumors, epididymal cystadenomas	< 10%
*EPAS-1 (HIF2A)*	Postzygotic/somatic	Multifocal sPGL (high penetrance) PCC (high penetrance)	NE	Pacak–Zhuang syndrome; Somatostatinoma; polycythemia; ocular lesions	29%
*IRP1*	Somatic	/	NE	/	/
*DLST*	Germline	sPGL (multiple)	NE	/	/
*SLC25A11*	Germline	/	NE	/	Increased risk of metastatic disease
*SUCLG2*	Germline	/	NE	/	/
Cluster 2 (kinase signaling)					
*RET*	Germline/somatic	PCC (high penetrance) usually bilateral (50-80%) sPGL (very low penentrance) HNPGL (very low penetrance)	E	MEN2 2A: medullary thyroid carcinoma, hyperparatiroidism, cutaneous lichen amyloidosis 2B: medullary thyroid carcinoma, multiple mucocutaneous neuromas, marafanoid habitus, intestinal ganglioneuromas	< 5%
*NF1*	Germline/somatic	PCC (low penetrance) sPGL, HNPGL (very low penetrance)	E and NE	Von Recklinghausen’s disease Café-au-lait spots, neurofibromas, Lisch nodules, Optic pathway/CNS gliomas, GIST	< 10%
*TMEM127*	Germline	PCC (intermediate penetrance) bilateral (33-39%) sPGL (low penetrance) HNPGL (very low penetrance)	E	PGL5 RCC	<5%
*MAX*	Germline/somatic	PCC (common bilateral) sPGL (very low penetrance)	E and NE	PGL7 renal oncocytoma; pituitary adenoma	7-25%
*HRAS*	Somatic	PCC PGL	/	/	/
*MET*	Germline/somatic	/	/	/	/
*MERTK*	Germline	/	/	/	/
*DNMT3A*	Germline	Multifocal HNPGL	/	/	/
Cluster 3 (Wnt signaling)					
*CSDE1*	Somatic	PCC	E	/	Increased risk of recurrent and metastatic disease
*UBTF-MAML3*	Fusion	PCC	E	/	Increased risk of recurrent and metastatic disease

PPGL, pheochromocytoma-paraganglioma; SDHA, succinate dehydrogenase complex flavoprotein subunit A; sPGL, sympathetic paraganglioma; HNPGL, head and neck paraganglioma; PCC, pheochromocytoma; NE, norepinephrine; DA, dopamine; NS, non-secreting; RCC, renal cell carcinoma; GIST, gastrointestinal tumor; SDHB, succinate dehydrogenase complex flavoprotein subunit B; SDHC, succinate dehydrogenase complex flavoprotein subunit C; SDHD, succinate dehydrogenase complex flavoprotein subunit D; SDHAF2, Succinate dehydrogenase complex assembly factor 2; FH, fumarate hydratase; MDH2, malate dehydrogenase 2; VHL, von Hippel-Lindau; EPAS-1, Endothelial PAS domain protein 1; HIF2A, hypoxia-inducible factor 2 alpha; IRP1, Iron Regulator Protein 1; DLST, Dihydrolipoamide S-Succinyltransferase; SLC25A11, Solute Carrier Family 25 Member 11; SUCLG2, Succinyl Co-A Ligase G2; RET, rearranged during transfection; E, epinephrine; MEN2, multiple endocrine neoplasia type 2; NF1, neurofibromatosis type 1; CNS, central nervous system; TMEM127, transmembrane protein 127; MAX, Myelocytomatosis-associated factor X; HRAS, Harvey rat sarcoma viral gene homologue; MET, Mesenchymal to Epithelial Transition; MERTK, Tyrosine Kinase Protooncogene; DNMT3A, DNA methyltransferase 3 alpha; CSDE1, Cold shock domain containing E1; UBTF-MAML3, Upstream Binding Transcription Factor Mastermind-like Transcriptional Coactivator 3.

Instead, cluster 2 tumors include neoplasms due to mutations of the neurofibromatosis type 1 (*NF1*) tumor suppressor gene, the rearranged during transfection (*RET*) proto-oncogene, genes encoding transmembrane protein 127 (*TMEM127*), MYC-associated factor X (*MAX*) and *HRAS*. Mutations of cluster 2 genes regard activation of kinase receptor signaling pathways, translation initiation, protein synthesis, and pathways involved in maintenance of neural/neuroendocrine identity (26).

Cluster 3 comprehends somatic *CSDE1* (Cold shock domain–containing E1 gene) mutations and *MAML3* (a member of the Mastermind-like family of transcriptional co-activators) fusion variants implicated in Wnt-pathway signal alterations (25).

The specific genetic background influences the biochemical and clinical phenotypes because the underlying mutation determines a variable expression of biosynthetic enzymes involved in the synthesis of catecholamines by the tumors (28). In more detail, cluster 1 neoplasms are typically characterized by noradrenergic phenotype, on the contrary cluster 2 tumors by adrenergic phenotype (29). Literature data showed that noradrenergic PPGLs typically manifest in younger age that adrenergic ones (30). Based on these assumptions, a study performed by Pamporaki et al. (9) on large cohort of pediatric and adult patients with PPGLs demonstrated, not only a childhood predominance of extra-adrenal, multifocal, metastatic, recurrent, and hereditary PPGLs, but also the link between these phenotypic features to a higher prevalence of noradrenergic and related cluster 1 hereditary tumors in pediatric than adult patients.

As mentioned above, the biochemical properties of PPGL are related to the underlying genetic mutations able to influence differentiation of tumor progenitor cells and consequently the secretory pathways and the epigenetic remodeling profiles (21).

The complex relationship between genotype and phenotype in PPGLs and the characteristics of the main tumor-susceptibility genes and their related hereditary syndromes are summarized in **Table 1**.

## Diagnostic work-up

Biochemical testing is recommended as the initial step in evaluation of suspected PPGL and should include plasma or 24-hours urinary free metanephrines measurement, performed using liquid chromatography assay (5, 8). Taking into account that 24-hour urine collection may not be feasible in young children, a plasma sample is often the initial biochemical test obtained in childhood. Indeed, two studies documented a high diagnostic accuracy of plasma free metanephrines for pediatric patients (31, 32). Pre-analytical considerations are specific challenges that impact upon the interpretation of biochemical tests in pediatric patients. In fact, sympathoadrenal activation triggered by upright posture, distress associated with venepuncture, emotional stress, as well as many medications (e.g. acetaminophen, tricyclic antidepressants, amphetamines,.) should be carefully evaluated. In general, normetanephrine or metanephrine values three-/four-fold or higher above the upper limit of the reference ranges require anatomical imaging for tumor localization and staging. Initial imaging studies include either contrasted enhanced computed tomography (CT) or magnetic resonance imaging (MRI), given their similar diagnostic performance (33). However, MRI is the preferred imaging modality for patients with head and neck PPGL and in those with metastatic disease and it is necessary if the initial imaging of the abdomen and pelvis does not identify the neoplasia. Moreover, functional imaging is a complementary technique useful for disease staging, detection of metastases or recurrent/multiple tumors. However, in childhood the indication for functional imaging must be carefully balanced against the radiation risk. The different functional imaging modalities include 68Ga-DOTATATE, 18F-fluorodopa (FDOPA), 18F-fluorodeoxyglucose (FDG) PET/TC and 123I-metaiodobenzylguanidine (123I-MIBG). The choice of the specific functional tracers should be guided by the known or suspected genetic mutation of the patient (34).

## Prevention and treatment of PPGL-induced hypertensive crisis

The most important way to approach catecholamine-induced hypertensive crisis is the prevention of their onset. Therefore, the first part of this section is dedicated to the preoperative medical management.

## Preoperative prevention of hypertensive crisis

To reduce the impact of hypertensive crises, potentially occurring during intraoperative manipulation of the tumor, adequate preoperative oral anti-hypertensive treatment is required.

From a pathophysiological perspective, a sequential approach with initial α-adrenergic blockade followed by β-adrenergic blockers is recommended to avoid reflex tachycardia (**Table 2**). Normalization of blood pressure and heart rate in pediatric patients requires longer treatment than in adults; therefore, compared with adults, it is recommended to start the treatment at least 14 days before surgery. This is probably related to the low-dose regimen and progressive titration of drugs to avoid side effects and to the fact that children are more sensitive to sympathetic overactivity (9, 11). In addition to antihypertensive therapy, adequate salt and water supplementation is required to avoid orthostatic hypotension related to volume contraction due to catecholamine release and drug side effects. After the initiation of α-blocker therapy, a supplementation of 6 to 10 grams per day of salt, depending on patient’s body surface area and an increase in fluid intake up to 1.5 times the weight-corrected levels are recommended.

**Table 2:** Preoperative management of PPGL in children (8–11).

		Drug	Mechanism	Dose	Side effects	Comments
**Pre** **operative** **(start 14 days prior to the surgery)**	First-line treatment	Doxazosin	Selective α1-blocker	1-2 mg/day in 1-2 doses; Max 4-16 mg/day	Orthostatic hypotension, dizziness, fatigue, drowsiness	No reflex tachycardia, long action (>24h)
		Prazosin		0.05-0.1 mg/kg/day in 3 doses Max 0.5 mg/kg/day (20 mg/day)		Less used
		Terazosin		1 mg/day; Max 20 mg/day		Less used
	Sequential approach	Phenoxy- benzamine	Non selective α1/α2-blocker	0.2-0.25 mg/kg/day (max 10 mg/dose) Max 2-4 mg/kg/day (60 mg/day)	Orthostatic hypotension, nausea reflex tachycardia, nasal congestion, central sedation, abdominal pain	Associate β blocker 3-4 days prior to the surgery
		Atenolol	Selective β1-blocker	0.5-1 mg/kg/day in 1-2 doses; Max 100 mg/day	Dizziness, Fatigue	Never give before α-blocker
		Metoprolol		1-2 mg/kg/day in 1 dose; Max 200 mg/d		
		Propranolol	Non selective β1/β2-blocker	1-2 mg/kg/day in 2-4 doses; Max 640 mg/day	Dizziness, fatigue, bronchoconstriction	
		Labetalol	α1/β-blocker	1-3 mg/kg/day in 2 doses; Max 1200 mg/day		
	Adjunctive agents	Amlodipine	Calcium channel blocker	0.05-0.1 mg/kg/day; Max 10 mg/day	Peripheral edema, palpitations, gingival hyperplasia, headache	Use as adjunct
		Metyrosine	Tyrosine hydroxylase inhibitor	20 mg/kg/day in 4 doses; Max 2500 mg/day	Lethargy, extrapiramidal symptoms, diarrhea, crystalluria	Never give before α-blocker
	Salt	6-10 g/day				
	Fluids	1.5 times maintenance fluids per kg				

The goal is to achieve a blood pressure within the 50-90th percentile for age, gender and height, aiming to obtain values closer to the 50th percentile in the very last preoperative days.

The most used drugs are long-acting non-selective α1/α2 receptor blockers such as phenoxybenzamine or long-acting selective α1 receptor blockers such as doxazosin, prazosin or terazosin. The most frequent side effect is orthostatic hypotension. Non-selective α1/α2 receptor blockers additional side effects also include reflex tachycardia, central sedation, nasal congestion, nausea and abdominal pain. Selective α1 receptor blockers generally have a better side effect profile, with no reflex tachycardia and more prolonged activity (>24 hours). The risk of postoperative hypotension with selective blockers is also low, therefore they are used preferentially in many centers.

Second-line or adjunctive agents include calcium channel blockers, such as amlodipine or nifedipine and tyrosine hydroxylase inhibitors, such as metyrosine. The hypotensive effect of calcium channel blockers is minor, and their use is usually limited when blood pressure control with selective and nonselective α-blockers is ineffective or in case of severe adverse effects. Metyrosine is used for short periods before surgery in combination with α-blockers to provide better pre- and intraoperative blood pressure control. Side effect profile include extrapyramidal symptoms, diarrhea, orthostatic hypotension, drowsiness, xerostomia, and neuromuscular symptoms, as well as crystalluria, therefore its use in pediatric age is limited.

When nonselective α-blockers are used, β-adrenergic blockers are often added a few days before surgery to relieve α-blockers-related reflex tachycardia. β-adrenergic blockers should never be started before α-blockers because catecholamine-related vasoconstriction would trigger a hypertensive crisis. Selective β1-adrenergic blockers, such as atenolol and metoprolol, are preferred over nonselective β1/β2-blockers (propranolol) because of the risk of bronchoconstriction. Another nonselective β-blocker agent, with combined selective α1-blockade is labetalol. The latter should not be used alone for the high risk of hypertensive crisis (8–11).

## General and intraoperative management of PPGL-induced hypertensive crisis

In case of hypertension urgency where children can tolerate oral therapy, intravenous administration may be initially avoided. However, hypertensive crises in children with PPGL occur predominantly during intraoperative manipulation of the tumor, even despite adequate preoperative treatment. Therefore, continuous invasive blood pressure monitoring by intra-arterial catheterization is essential during surgery to help anesthesiologists in assessing blood pressure fluctuations (8). In case of intraoperative hypertensive crisis, intravenous administration of short-acting antihypertensive drugs is mandatory. These indications can be also applied to the general management of hypertensive crisis out of the surgical setting.

From a pathophysiological perspective, as mentioned earlier, the drugs of choice to treat PPGL hypertensive crises are α-blockers, such as urapidil, a combined peripheral selective α1 receptor antagonist and central serotoninergic 1A receptor agonist, or phentolamine, a non-selective α1/α2 receptor antagonist. Urapidil is particularly useful, as it does not induce reflex tachycardia and is not associated with alterations of the renin-angiotensin-aldosterone system, thereby minimizing side effects. However, there are limited case series on the use of this drug in the pediatric population (14, 35). One of these is a multicentric Italian retrospective survey on treatment of hypertension in children with neuroblastoma. Intraoperative hypertension management was somewhat dissimilar among the participating centers, apart from a certain consistency in the intraoperative use of urapidil (36). Side effects associated with α-blockers in PPGL patients are rare and generally not severe. The main side effects reported in literature include excessive hypotension, tachycardia, dizziness, and nasal congestion (8, 10, 37).

In cases of hypertensive crises accompanied by tachyarrhythmias, when α-blockade is achieved, short-acting intravenous β-blockers may be utilized in combination, including labetalol, an α1-β blocker with a 1:7 ratio for intravenous administration, and esmolol, a selective β1-antagonist (10).

Although the majority of experience with esmolol, especially in little children younger than 6 years, is in management hypertension during and after intervention for aortic coarctation repair (38). Romero et al. (39) in a retrospective analysis of medical records of 10 children (from the age of 6 months to 18 years), that were diagnosed with a catecholamine secreting tumor from 2005–2013 and underwent surgical removal, showed how 80% of these patients experimented hypertension crises during surgery and most of them were treated with esmolol, labetalol, but also sodium nitroprusside. The disadvantage is the same as in all β-blockers, namely its negative inotropic effect and potential for bronchoconstriction. Consequently, it should be avoided in children with asthma or those suffering from decompensated or unstable congestive heart failure (14). The principal side effects of this class of drugs are dizziness, bradycardia and risk of atrio-ventricular block (10).

Second-third line treatments for hypertensive crises in children with PPGL include calcium-channel blockers, particularly second-generation dihydropyridines such as nicardipine, and nitroderivatives. Among these options, sodium nitroprusside, a preferential arterial vasodilation, is the drug of choice, because nitroglycerin, reducing cardiac preload by venous vasodilation, can potentially induce significant reflex tachycardia (10, 14). There is notable experience in using nicardipine for managing severe hypertension in children (40, 41). The main side effects observed were related to its vasodilatory effects, including tachycardia, flushing, palpitations, and hypotension. Sodium nitroprusside treatment provides additional benefits for controlling coronary vasospasm. However, it requires careful monitoring due to the potential risks of cyanide and thiocyanate toxicity. In addition, precautions must be taken to protect it from exposure to light (10, 14). Finally, in children with hypertensive crises due to PPGL, as an adjunctive therapy, treatment with dexmedetomidine, a central α2-agonist, and magnesium sulphate, a vasodilator that also inhibits catecholamine release from the adrenal medulla and sympathetic nerve endings, has been described (8, 10). In particular, magnesium sulphate is a valid and safe alternative for the pediatric population and is also suitable for use in pregnant women (42, 43). The main side effects of dexmedetomidine include respiratory depression and bradycardia. Conversely, magnesium sulphate should be used with caution in individuals with neuromuscular disorders due to the risk of paralysis (8, 10). Details about all these treatments are described in **Table 3**. In case of intraoperative hypotension, intravenous infusion of crystalloid or colloid fluids and administration of vasoactive agents may be necessary, with particular awareness in case of catecholamine-induced cardiomyopathy, as these patients are at risk for pulmonary edema secondary to volume overload.

**Table 3:** Management of hypertensive crises induced by catecholamine secreting tumors in children (8, 10, 11, 14).

Drug	Mechanism	Dosage	Side effects	Contraindications
FIRST LINE TREATMENT				
Urapidil	Selective α1-adrenergic receptor antagonist - central serotoninergic 1A receptor agonist	Initial 0.5–4.0 mg/kg per hour Maintenance 0.2–2.0 mg/kg per hour	Hypotension, tachycardia, dizziness, central sedation, nausea and nasal congestion	Athero-venous shunt, stenosis of the aortic isthmus
Phentolamine	Competitive non selective α1/α2 adrenergic receptor antagonist	Bolus 0.1-5 mg/Kg	Hypotension, tachycardia, dizziness, central sedation, arrythmias and nasal congestion	//
Labetalol (in case of concomitant tachyarrhythmias, after adequate α-adrenergic blockade)	Combinate α1/β-adrenergic blocker (ratio 1:7)	0.25–3 mg/kg/hour Titrate slowly Max: 3 mg/kg/hour	Orthostatic hypotension, dizziness	Asma, sinuses bradycardia, atrio-ventricular block, heart failure
Esmolol (in case of concomitant tachyarrhythmias, after adequate α-adrenergic blockade)	Selective β1-adrenergic blocker	Bolus of 500–600 μg/kg over 2 min. Maintenance 200 (50–250) μg/kg/min. Max 500 µg/Kg/min	Hypotension, bradycardia, risk of atrio-ventricular block	Asma, sinuses bradycardia, sick sinus syndrome, atrio-ventricular block, hypotension, heart failure, cardiogenic shock, pulmonary hypertension
SECOND LINE TREATMENT				
Sodium nitroprusside	Vasodilator (nitro-derivates)	Starting: 0.3–0.5 µg/kg/min. Titrate by 0.1 µg/kg/min every few minutes. Max: 10 µg/kg/min	Tachycardia, flushing, palpitations, and hypotension. Monitor for risk of cyanide and thiocyanate toxicity (so protect from light)	Renal and/or hepatic failure, hypothyroidism, deficit of vitamin B12
Nicardipine	Dihydropyridine calcium channel blocker	Starting: 0.5–1 µg/kg/min. Max: 4–5 µg/kg/min	Tachycardia, flushing, palpitations, and hypotension, edema, headache	Pathological hyperlipemia, nephrosis or acute pancreatic inflammation secondary to hyperlipemia
OTHER TREATMENTS				
Magnesium sulphate	Vasodilator, inhibits catecholamine release from adrenal medulla and sympathetic paraganglia	Loading dose: 40–60 mg/kg over 10 minutes. Maintenance: 15–30 mg/kg/hour	Neuromuscular paralysis	Use with caution in those with neuromuscular disease (risk of paralysis)
Dexmedetomidine	Central α2-agonist	Loading dose: 0.5–1 µg/kg/dose over 10 minutes. Maintenance: 0.2–0.5 µg/kg/hour	Respiratory depression, bradycardia, xerostomia	In those with reduced respiratory drive

## Postoperative complication management

Continuous monitoring in the first 48 hours after surgery is indicated for a high risk of hemodynamic instability. Multifactorial hypotension, responsive to colloid/crystalloid infusion, may occur due to the downregulation of adrenergic receptors and acute withdrawal of catecholamines after surgical removal of the mass, as well as the prolonged action of antihypertensive agents used in the preoperative period and the short-term effect of intraoperative management. Hypoglycemia may also occur postoperatively due to rebound hyperinsulinemia, which results from the loss of the inhibitory action of catecholamines on pancreatic β-cells. This condition can be treated with the infusion of glucose-containing fluids (8, 10).

## Conclusions

Although hypertensive crises in children are rare conditions, the precise determination of their prevalence is more challenging than in adults, due to the heterogeneity in its definition. Catecholamine excess represents a rare cause of hypertensive crisis, the management of which is complex and requires careful selection and timing of therapy administration, even more in pediatric patients. Therefore, it would be advisable to manage these cases in tertiary care centers through interdisciplinary collaboration involving endocrinologists, pediatric intensivists, anesthesiologists, nephrologists, emergency physicians and surgeons to optimize therapeutic success (8–10).
